# An exploratory study of Clinical and Translational Science Award community-engaged research training programs

**DOI:** 10.1017/cts.2018.23

**Published:** 2018-06-25

**Authors:** Linda Ziegahn, Lucinda Nevarez, Thelma Hurd, Jill Evans, Yvonne Joosten, Jill Dumbauld, Milton “Mickey” Eder

**Affiliations:** 1 Community Engagement and Research Program, Clinical and Translational Science Center, University of California Davis School of Medicine, Sacramento, CA, USA; 2 Social Work Department, College of Public Policy, University of Texas at San Antonio, San Antonio, TX, USA; 3 Department of Surgery, University of Texas Health Science Center at San Antonio, UT School of Public Health-Houston, San Antonio Extension, San Antonio, TX, USA; 4 Center for Population Health Sciences, Stanford University School of Medicine, Stanford, CA, USA; 5 Office for Community Engagement, Vanderbilt Institute for Medicine and Public Health, Vanderbilt University School of Medicine, Nashville, TN, USA; 6 Education, Training and Career Development Program, Clinical and Translational Research Institute, University of California San Diego, La Jolla, CA, USA; 7 Department of Family Medicine and Community Health, Clinical and Translational Science Institute, Office of Community Engagement to Advance Research and Community Health, University of Minnesota, Minneapolis, MN, USA

**Keywords:** Community-engaged research, community engagement, training, academic medical education, health disparities

## Abstract

**Background:**

The Clinical and Translational Science Award (CTSA) institutions are increasing development of training programs in community-engaged research (CEnR) to support translational science.

**Methods:**

This study sampled posters at CTSA national meetings to identify CEnR training approaches, topics, and outcomes.

**Results:**

Qualitative analysis of 30 posters revealed training topics and outcomes focused primarily on CEnR capacity building, overcoming barriers, systems change, and sustainability.

**Conclusion:**

Further research should focus on development and results of CTSA CEnR training program metrics.

## Introduction

As the Clinical and Translational Science Award (CTSA) enterprise has matured, training programs for health researchers have received increasing attention [1–3]. An essential part of this training has been in community-engaged research (CEnR), a multifaceted process aimed at translating clinical science from bench to patients to communities [4]. CEnR has been documented as an effective method for not only engaging communities in research but also for planning, evaluating, and disseminating research results [5]. Although many CTSA funded institutions have developed CEnR training programs for trainees, faculty, medical students, community members, and other potential health research partners, little is known about the goals, strategies, and outcomes that indicate program success [5, 6]. Knowledge of processes such as trust-building, research ethics, partnership formation, and research design and evaluation is essential for understanding how to best measure and support the workforce that sustains CEnR and more broadly, the translational science mission.

Staff across the CTSA consortium have developed innovative approaches to train researchers at all levels on the bidirectional mission of CEnR and to close gaps between experimental research and evidence-based practice [7–10]. Many of these approaches rely on community partnerships while emphasizing capacity building to sustain CEnR within academic medical contexts and communities [8, 9, 11]. To date, programmatic motivations, strategies, and outcomes of CEnR training have not coalesced into a shared approach across the CTSA consortium. The Institute of Medicine report on CTSA conduct during the first 2 funding cycles characterized community partner training as “rather informal” [6] and advocated for learning cultures focused on experiential learning, team science, leadership, community engagement, entrepreneurship, and dissemination of results. CTSAs further expressed interest in developing metrics to assess bidirectional partnership aims to enhance community research capacity and infrastructure [12–19].

This descriptive study sought to provide a snapshot of how CTSA institutions addressed workforce development across the CEnR mission. We aimed to reveal some of the innovative and responsive approaches of CEnR researchers and their community partners to education that will ultimately bring together the distinct yet complementary knowledge bases of scientists and community members. Research questions guiding this study are as follows:

- How did CEnR training reflect the basic principles of CEnR?
- What issues (e.g., health, CEnR processes, health disparities) motivated training?
- What key training topics were deemed important by CEnR participants?
- What were the perceived outcomes of CEnR training?

## Materials and Methods

### Background

Between 2009 and 2014 the National Center for Advancing Translational Sciences sponsored annual CTSA Community Engagement meetings in Bethesda, MD. Participants from CTSA academic medical institutions and select community partners met to discuss models, advances, metrics, best practices, and future directions in CEnR. The meetings included poster sessions where attendees shared innovative projects, including those dedicated to training researchers and community members in CEnR knowledge and skills. Study authors, all members of the CTSA Community Engagement, Education, Training, and Scholarship working group, determined study goals and criteria for poster inclusion, developed an interview protocol and guide, conducted interviews, and analyzed data.

### Sample Selection

All posters presented at the 2012–2014 CTSA community engagement meetings that highlighted CEnR education or training to further health research were included in a convenience sample. Poster inclusion criteria were defined as instructional endeavors involving CTSA CEnR program staff and selected academic, clinical, or community partners, aimed at educating researchers and/or community members on goals, methods, and applications of CEnR. Out of a total of 175 posters for the 3 years, 30 (17%) CEnR posters/projects met the eligibility criteria and lead authors of each poster agreed to participate in the study (see Table 1).

**Table 1:** Posters in study related to community engagement training and education

	2012	2013	2014	Total
Total posters	55 (100%)	60 (100%)	60 (100%)	175 (100%)
CEnR posters selected	22 (40%)	33 (55%)	22 (37%)	77 (44%)
Poster contacts not responsive	12 (22%)	13 (22%)	16 (27%)	41 (23%)
Interview sample identified	10 (18%)	20 (33%)	6 (1%)	36 (21%)
CEnR not part of poster project	3 (5%)	3 (5%)	0	6 (3%)
Interviews included in study	7 (13%)	17 (28%)	6 (1%)	30 (17%)

CEnR, community-engaged research.

### Qualitative Interview Guide and Process

Team members collaboratively designed and pretested a 17-item interview instrument to identify real-world health issues that informed CEnR training project focus, audiences and goals, the range of educational formats and evaluation measures used, and perceived project outcomes. An interview guide was developed and uploaded along with the survey instrument into Vanderbilt University’s REDCap database to allow the entire study team access. This survey contained both forced choice and open-ended question formats to enable respondents to candidly explain interrelated training needs and strategies. Each respondent participated in a 45-minute phone interview. Interviewers either typed responses immediately or from phone call notes into the REDCap template.

### Data Analysis

Qualitative and quantitative interview data were downloaded from REDCap into an Excel spreadsheet. The 7-member research team broke into smaller groups to analyze open-ended interview responses, following the qualitative data analysis and intercoder reliability guidelines recommended by Bernard and Ryan [20]. Frequencies and percentages from yes/no and forced choice questions were calculated using the SPSS21 software package.

## Results

### Profile of Poster Projects

While the CEnR training projects represented by the 30 posters we sampled varied in instructional formats and activities, they all sought to further community engagement in translational research [19]. Community members included members of underrepresented groups and lay research advisors, healthcare providers ranging from physicians to community health workers, and representatives of community-based organizations. Health researchers engaged in training projects included faculty, research staff, postdoctoral fellows, graduate and undergraduate students.

Twenty-three (77%) of the 30 projects were designed to train both community members and health researchers so that they could jointly conduct CEnR projects, reflecting variations of the “train the trainer” model. Examples included training high school teachers in community-based participatory research methods so that they could then train students and families as partners in community-identified health research projects. In another case, *promotoras* were trained in CEnR methods, project management and collection of evaluation data to lead community cardiovascular health sessions. Faculty and/or community members often alternated training roles, exemplifying the shared expertise tenet of CEnR. Five (17%) CEnR training projects engaged community members only, and 2 (7%) were aimed uniquely at researchers.

Respondent motivations to launch CEnR educational programs included health specific problems (e.g., cancer, cardiovascular disease, childhood asthma), and/or need for capacity building skills and knowledge to create effective CEnR partnerships focused on reducing health disparities. Training topics addressed current research on disease prevention or treatment, knowledge and skills necessary for building CEnR infrastructure, ethical research conduct, and bidirectional communication between community members and academics. CEnR motivations and training topics are displayed in Table 2.

**Table 2:** Community-engaged research (CEnR) training design motivation and resulting topics

Motivations	n (%)		Training topics	n (%)
Specific community health issues	10 (33%)		Building CEnR infrastructure	11 (37%)
Increasing academic CEnR skills	6 (20%)		Ethics and the IRB	11 (37%)
Increasing community CEnR skills	5 (17%)	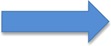	Preventive health topics	10 (33%)
Developing academic-community partnerships	3 (10%)		Grant writing and research methods	8 (27%)
Increasing underrepresented populations in clinical trials	2 (7%)		Health research topics	4 (13%)
Other motivations*	5 (17%)		Translational research	2 (7%)
			Science communication	2 (7%)
			Health disparities	2 (7%)

IRB, Institutional Review Board.

* Increasing community scientific literacy, including community members in planning research infrastructure, promoting shared understanding of research language and health priorities.

### Training Outcomes

In total, 25 projects (83%) reported conducting formative evaluations of CEnR training outcomes. However, we also asked the question “What happened as a result of this project/course?” in order to get at respondents’ broader impressions of changes resulting from training. We analyzed references to outcomes in other questions as well. Respondents’ characterizations of the benefits and challenges resulting from their training programs fell into 7 primary categories, displayed in Table 3.

**Table 3:** References to outcomes of community engagement education and training

CEnR training outcome	Number of references	Percentage*
Capacity building	63	65.6 (n=96)
∙ Team strengthening	18	28.6 (n=63)†
∙ Social capital	13	20.6 (n=63)
∙ Empowerment	11	17.5 (n=63)
∙ Awareness and understanding	7	11.1 (n=63)
∙ Trust building	7	11.1 (n=63)
∙ Infrastructure	5	7.9 (n=63)
∙ Innovation	2	3.2 (n=63)
Barriers	9	9.4 (n=96)
Systems change	8	8.3 (n=96)
Sustainability	8	8.3 (n=96)
Dissemination	6	6.2 (n=96)
Tools and resources	5	5.2 (n=96)
Preventive health topics	4	4.2 (n=96)

CeNR, community-engaged research.

* Totals will not add up to 100 due to rounding.

† Totals are based on a subpopulation of the 63 references to capacity building as an outcome.

#### Capacity Building

Two-thirds of poster authors referred to capacity building—in particular, team building, social capital, and empowerment—as an outcome. Community-researcher teams generated co-learning, common team identity, and bidirectional exchanges of knowledge and skill acquisition. Other capacity building outcomes included increased community participant confidence in formulating research questions, participation in hospital research coalitions and government policy and advisory boards, and advising researchers on recruitment and study design. Enhanced social capital examples included greater confidence in community-researcher interactions, community investment in research, professional growth, project ownership, and research networking.

Respondents further reported learning how collective community support could address population health challenges and development and expansion of CEnR infrastructure at the community, faculty and academic institutional levels. Examples included the creation of a community research review board, a new process for disseminating research findings to communities, and increased support for community-identified health issues such as youth trauma and grief counseling, and radon testing and mitigation. Innovations mentioned included researchers learning from community members about the value of metaphors for science communication. Finally, several respondents mentioned trust as either a key issue, project goal, or result of building community partnerships.

#### Barriers

Respondents identified inadequate time for community-researcher interaction, scheduling conflicts, board turnover and retention of community members as challenges to achieving desired CEnR training outcomes.

#### System Change and Policy

CEnR training projects that catalyzed system or policy changes, such as the science café project, were perceived as strengthening institutional buy-in of communities as research partners by researchers and clinicians. One CTSA institution required community engagement training for faculty and students as a result of training, and another established pilot funding for projects with health centers.

#### Sustainability

Respondents credited increased interest in conducting CEnR research, obtaining formal CEnR training, or continuing projects at the conclusion of funding to newly learned skillsets. In some cases, new community partnerships were established as a result of training, and existing partnerships were leveraged to develop or replicate new projects through funding from the Patient Centered Outcomes Research Institute. However, several participants deemed institutional support for coalition activities over the project lifespan insufficient.

#### Dissemination

Approaches to disseminating knowledge and skillsets to community members and organizations and institutions included train the trainer workshops, publications, poster presentations, and community forums.

#### Resources and Tools

Community resources and tools produced through CEnR partnership training included online guides and curricula available through community portals, community partner registries to enhance advisory board memberships and sustain projects, and innovative teaching methods to impart translational science knowledge to lay communities.

#### Preventive Health Topics

Respondents attributed specific preventive health interventions and behaviors to CEnR training of community members. For example, screening men for colon cancer and weight reduction through obesity research and prevention training projects aimed at sharing health behavior strategies with families.

## Discussion

This study adds detailed information to the CEnR literature on instructional rationales, audiences, strategies, and training outcomes developed by CTSA community engagement staff. Collectively, these approaches imply a set of best practices in CEnR, summarized as follows: first, CTSA-affiliated health researchers—along with their community partners—engaged in the boundary spanning roles and behaviors necessary to connect the academic experience of researchers with the lived experience of community members. Health researchers and community partners reported developing equitable research collaborations through bidirectional communication in the design and implementation of training [19]. Alternating and overlapping training sessions for academic and community audiences demonstrated commitment to applying CEnR principles to real-world settings [8].

Second, capacity building skills in research design processes and phases as well as community partnership development strategies were key motivators for CEnR program development [7–11, 14]. These motivations translated into topics that reflected the relational, ethical, research skill-based, and dissemination aspects of CEnR. The “train the trainer” emphasis for building CEnR skills in these early years of the CTSA consortium coincided with the CTSA Collaboration/Engagement Task Force and Workforce Development workgroup focus on collaboration skills for both translational researchers and community partners [9]. Community interest in addressing specific diseases was apparent in projects dealing with prevention and chronic illness.

Third, perceived outcomes included not only a range of capacity building skills, but also concrete training programs, dissemination strategies, and changes in CTSA funding priorities aimed at strengthening institutional commitment to CEnR and its role in translational science.

## Limitations

The study convenience sample makes no claim to represent all CTSA institutions or those academic institutions or nonprofit organizations where CEnR training might occur. However, the posters were presented at NIH funded national meetings after undergoing anonymous peer review and selection.

Because of the scope of translational science, results yielded a variety of training topics, audiences, and formats, making comparisons across training programs precarious. Further, the nonevaluative nature of study design meant that program outcomes were based on participant perspectives and not independently measured.

## Future Directions

This study illustrates trends in CEnR training that merit more rigorous inquiry and evaluation. A more systematic inventory of CTSA CEnR training activities would provide a more complete picture of metrics used to assess training program development and outcomes. Additional research should address the adequacy of CTSA CEnR infrastructure and sustainability of successful projects:

- What was the role of the community and partnerships in metrics development for training?
- How were CEnR skills and knowledge gained through training implemented in CEnR programming or other translational science efforts?
- How can evaluation and input from participants be used to improve training and advance project development?
- What types of training activities increase the numbers and success of academic-community research partnerships?
